# Heart Failure in South Asia

**DOI:** 10.2174/1573403X11309020003

**Published:** 2013-05

**Authors:** Harikrishnan Sivadasan Pillai, Sanjay Ganapathi

**Affiliations:** 1 Department of Cardiology, Sree Chitra Tirunal Institute for Medical Sciences and Technology, Trivandrum, Kerala, India

**Keywords:** Heart failure, South Asia, India, epidemiology, aetiology, prevention, heart failure clinics.

## Abstract

South Asia (SA) is both the most populous and the most densely populated geographical region in the world. The countries in this region are undergoing epidemiological transition and are facing the double burden of infectious and non-communicable diseases.

Heart failure (HF) is a major and increasing burden all over the world. In this review, we discuss the epidemiology of HF in SA today and its impact in the health system of the countries in the region. There are no reliable estimates of incidence and prevalence of HF (heart failure) from this region.

The prevalence of HF which is predominantly a disease of the elderly is likely to rise in this region due to the growing age of the population. Patients admitted with HF in the SA region are relatively younger than their western counterparts. The etiology of HF in this region is also different from the western world. Untreated congenital heart disease and rheumatic heart disease still contribute significantly to the burden of HF in this region. Due to epidemiological transition, the prevalence of hypertension, diabetes mellitus, obesity and smoking is on the rise in this region. This is likely to escalate the prevalence of HF in South Asia.

We also discuss potential developments in the field of HF management likely to occur in the nations in South Asia. Finally, we discuss the interventions for prevention of HF in this region

## EPIDEMIOLOGY OF HF IN SOUTH ASIA

South Asia is both the most populous and the most densely populated geographical region in the world. The South Asian (SA) countries which include India, Pakistan, Bangladesh, Sri Lanka, Bhutan, Maldives and Nepal (as per the World Bank) is the home to one-fifth of the world population. The countries in this region are undergoing epidemiological transition and are facing the double burden of infectious and non-infectious diseases [1]. 

Heart failure is a major and increasing burden all over the world [2]. There are no reliable estimates of HF (heart failure) incidence and prevalence in this region. There are only some projections based on prevalence data from Western countries [3, 4].

In this review, we discuss the epidemiology of HF in SA today and its impact in the health system of the countries in the region. We also discuss potential developments in the field of HF management likely to occur in these nations. Finally, we discuss the interventions for prevention of HF in this region.

There is no reliable data regarding the incidence or prevalence of HF in South Asia or indeed in Asia [3, 4]. Mendez and Cowie reported lack of population based HF studies even in the whole developing world, as in 2001 [5]. The 2008 Scientific statement on the prevention of HF from the American Heart Association mentions that such data does not exist except in US and Europe [6].

HF is predominantly a disease of the elderly, as the lifetime risk for HF increases with age [2], so the burden of HF is likely to rise with the growing age of the population in SA (Table **1**). The number of people above 60 years of age in the region is projected to increase from 133 million in 2011 to 494 million in 2051.

Pais*, et al*. projected the prevalence of HF in India based on the rates in the US [4]. Based on the same assumptions and using 2010 data (HF Prevalence USA—5 800 000 [7] out of a total population of 308,745,538), we reach a prevalence of 1.87%. If we apply this prevalence rate in the Indian population of 2011 [8], i.e., 1.21 billion, the number of patients with HF is 22.7 million. If we extrapolate to the whole of SA (total population of 1.63 billion in 2011), assuming a uniform epidemiological pattern in the whole region, the prevalence of HF is 30 million.

Huffman and Prabhakaran have estimated the prevalence of HF in India, based on approximate prevalence estimates (from India) and mortality data derived from the western literature3. They have considered the prevalence estimates for the year 2000 from India and used the yearly HF incidence rates for patients with coronary heart disease (CHD) and suggested that every year, 120 000-690 000 Indians could develop symptomatic HF secondary to CHD. Therefore, assuming 50% mortality at 5 years in those with HF [7], (“assuming none has HF at baseline and the at-risk population does not diminish”) they have estimated the prevalence of HF after 5 years. These estimates are given in Table **2**.

Considering the fact that the treatment patterns and thus the mortality may be totally different in SA compared to the west, these calculations (as the authors themselves concede) have limitations, but these are the only estimates available to us.

The other two major disease groups contributing to HF in the SA region are congenital heart disease and rheumatic heart diseases (RHD). Based on 2001 population data, assuming a lowest estimate of 0.21%, the number of patients with RHD in the age group 5-40 years in India is calculated to be about 1.4 million [17]. The incidence and prevalence of HF in RHD are unknown. Therefore it is not possible to accurately estimate the contribution of RHD to the burden of HF, but it is likely to be substantial.

Huffman and Prabhakaran report that the estimated prevalence of HF in India, due to CHD, hypertension, obesity, diabetes and RHD alone in 2000 ranges from 1.3 million to 4.6 million, with an annual incidence ranging from 491,600 to 1.8 million [3].

Similarly, assuming an incidence of 4/1000 live births for congenital heart diseases, the estimated number of children (0-15 years) with established congenital heart disease based on the 2001 census in India is 1.41 million [17]. About one-third of these patients require long-term follow-up into adult life [17]. So the number of patients with significant congenital heart disease in adult life in India is likely to be very large. US data show that the probability of HF continues to increase with age in adult congenital heart disease and represents nearly one-quarter of all mortality [18].

Another notable factor is the comparison of age of the patients with HF in the region and western populations. Patients admitted with HF in the SA region are relatively younger than their western counterparts. The mean age of patients among 276 patients admitted with HF in 2008 in Pakistan was 54.4 [19], compared to the mean age of 73.1+/-13.9 years in a group of 500,000 US patients who were part of a “real world registry” [20].

The two large studies published on acute coronary syndromes (ACS) from India also show that the age of presentation of patients with acute coronary events is much earlier than the western population. The larger of the two studies is the Kerala ACS registry (25748 patients) [21]. The mean age of the patients in that study was 60.4+/- 12.1 years. The mean age of the patients in the CREATE (Treatment and outcomes of acute coronary syndromes in India ) registry of 20468 patients was 57.5+/- 12.1 years [22]. Both these registries have proven that Indian patients with HF secondary to acute coronary events are younger than their western counterparts.

The data from patients admitted at our Institute with acute HF in the year 2011 were analyzed - Table **3** (unpublished data). There were 105 admissions of whom, 35 were females. The mean (+/- SD) ages were 46.2 (14.4) years for males and 51.3 (15.4) years for females. 

## AETIOLOGY OF HF IN SOUTH ASIA

The aetiology of HF in India has likely changed in the last 50 years. Vakil and colleagues [23] (1949) reported that, the primary causes in 1281 hospitalized HF patients were represented by hypertension-coronary (31%), RHD (29%), syphilis (12%), and pulmonary (9%) groups.

There are no major studies describing the aetiology of HF from India. In a small study of 125 patients from India (1999), rheumatic heart disease was the commonest underlying heart disease (52.8%) followed by ischaemic and/or hypertensive heart disease (27.2%) [24]. In a study from Pakistan of 196 patients (2007) with systolic HF, 77% was due to ischaemic heart disease [25].

In western countries, there is overwhelming predominance of various cardiovascular risk factors as the contributory risk factors for HF. Follow-up data from the Framingham cohort in patients with HF showed a prevalence of hypertension in 70% (males) and 78% (females), CHD in 59% and 48% and valvular heart diseases in 22% and 31% respectively. A changing trend among the various aetiologies in the same cohort was noted, with significant increase in the prevalence of diabetes and CHD and a declining prevalence of hypertension and valvular heart diseases over a period of three decades [26]. Non-ischaemic dilated cardiomyopathy is an important contributor to acute HF, and according to the Acute Decompensated Heart Failure National Registry (ADHERE) registry, 47% of HF admissions were due to this entity [27].

By comparison, in sub-Saharan Africa, the main etiology of HF was rheumatic heart disease (32%), followed by dilated cardiomyopathy (25%) and hypertensive heart disease (17%). It is worth noting that CHD represented only 2% of the patients [28, 29].

Table **3** shows data from a tertiary heart care centre in South India (unpublished data) regarding the various aetiologies in patients admitted with HF. This picture is significantly different from the western literature.

There are many publications regarding HF in South Asians who have migrated to and are living in the developed nations. From these studies we can see that there are significant differences between HF in South Asians compared with other ethnic groups. The main differences as reported by Shantsila and colleagues [30] are provided in Table **4**.

In addition to the risk factors and etiology of HF mentioned above, there are other causes of HF which produce significant burden on the health systems of SA countries, including pulmonary hypertension due to various aetiologies, infective endocarditis and peri-partum cardiomyopathy. Anaemia which is very common in this region especially in females [36], can contribute to the burden of HF and also can influence the prognosis. Emerging infectious disease epidemics also contribute to the burden, an example is the leptospirosis outbreak in Sri Lanka where 4% of the cases were complicated by HF [37]. There are also rare cardiomyopathies in specific regions such as endomyocardial fibrosis(EMF) in Kerala, India, the prevalence of which is probably on the decline [38].

Pulmonary hypertension (PH) is a common cause of HF in this region considering the prevalence of RHD and COPD (chronic obstructive pulmonary disease). Pulmonary arterial hypertension occurs in about 70% of RHD [39]. Based on the prevalence estimates of RHD by Grover and colleagues [17], we can calculate that 0.9 million of the population in India is likely to suffer from PH associated with RHD. A significant percentage of these patients can be expected to develop HF during the course of their illness.

From a study of 35295 adult subjects (over 35 years of age) from India, COPD were diagnosed in 4.1% [40]. In a study of COPD hospital admissions in China, HF contributed in 19.6% of cases [41]. If we assume similar figures, burden of HF contributed by COPD in India may be very large.

## HFPEF - HEART FAILURE WITH PRESERVED EJECTION FRACTION

Heart failure with preserved left ventricular ejection fraction (HFPEF) is an increasingly recognized entity. HFPEF can have outcomes as poor as those associated with HF and reduced LVEF (left ventricular ejection fraction), but it does not yet have a proven effective management strategy [42]. Data from the US shows that as the awareness about this entity is improving, it currently represents >50% of HF cases [42-44]. In another study which recruited patients from Latin America, Middle East and North Africa, HFPEF showed an overall prevalence of 65%.

Compared to the patients with HF and a reduced ejection fraction, those with HF-PEF were more likely to be older, female and obese. They more often had a history of hypertension and atrial fibrillation and less frequently had a history of myocardial infarction [45].

We also know that the Indian subcontinent is witnessing young age escalation of CHD and its risk factors [46, 47]. The large burden of undiagnosed and under-treated hypertension along with diabetes and CHD are likely to escalate the incidence and prevalence of HFPEF in this region.

## ECONOMIC IMPACT OF HF IN SOUTH ASIA

As discussed, HF has a high prevalence in South Asia, affecting both the young and the elderly. Despite advances in therapy and management, HF continues to have high mortality as reported from the west [42]. Based on the Framingham Heart Study, 30-day mortality of HF is around 10%, 1-year mortality is 20-30%, and 5-year mortality is 45-60% [48]. The life-time risk of development of HF in Whites has been calculated in the Framingham study to be 20%2.

Western data show that HF causes high mortality or disability resulting in significant economic loss. In a study from Belgium (2001), there were a total of 19,398 admissions with HF as a primary diagnosis. The mean in-hospital stay for HF was 14.8 days and the total in-hospital cost of HF represented 1.8% of total hospital expenditure [49].

From a nationwide German database of more than two million insured people in 2002, 86,193 patients had a diagnosis of HF. The various health insurance companies paid 2.3 times more for patients with HF than without congestive HF. Moreover, costs for drugs were three times higher (1073 Euro vs. 366 Euro). This analysis clearly demonstrates the increased costs incurred for patients with congestive HF compared to other diseases [50].

In a real world US registry, the mean length of hospital stay was 8.7+/-28.6 days, and in-hospital mortality was 7.1%. Mean hospital cost per admission was USD 18,667 [20].

Overall, chronic HF consumes 1-2% of the total healthcare resources in the developed countries [51]. These data clearly show that HF causes significant burden to healthcare systems, consuming more resources than other diseases. Since the age of patients with HF in SA is much lower than that in the West and with the population at risk being higher, the economic impact would be enormous. The loss of the breadwinner of the family at a young age, given the high mortality of the disease will have a significant impact on the family, the society and ultimately the region.

We do not have DALY (Disability adjusted Life Years) estimates for HF. But we have the data revealing that DALY lost secondary to CHD in India have been predicted to increase from 7.7 million to 14.4 million in men and 5.6 million to 7.7 million in women from 2000 to 2020 [9]. So the burden of HF and the resultant DALY is also very likely to increase in the coming years. In addition to mortality, HF adds significantly to morbidity. For example cognitive function is highly impaired in decompensated HF patients, which improves but does not normalize after compensation [52].

As discussed earlier, the treatment of HF is too resource-consuming. The developed world spends significantly to manage these patients. But the resource-poor countries in SA cannot afford such expensive treatments. The majority of the people have to indulge in OOP (out-of-pocket health spending) in the South Asia region. For example the OOP expenditures for CVD (cardiovascular disease) as a proportion of overall OOP expenditures in India is 90% compared to 23% in the USA [53]. Huffman et.al have reported that following a first episode of ACS, catastrophic health spending occurred in 92% of low-income households in India, while distress financing was up to 64% [54]. HF also will have the almost the same impact or more with its higher morbidity and mortality.

## ISSUES IN MANAGEMENT OF PATIENTS WITH HF IN SOUTH ASIA

There are two major issues related to HF management in this region: accessibility and affordability.

South Asian patients have uneven and limited access to healthcare. The people living in remote villages, high lands and islands in this region often have limited access to a health care facility. In the towns, even if they have access, affordability is an issue. The public health care system in this region is overloaded, making access difficult to these patients.

Emergency medical services are not widely available in countries like India. For example Prabhakaran *et al*. report that patients who experience acute cardiac events, such as ACS, typically have longer symptom-to-door and door-to-needle times than in other countries [55].

Xavier *et al*. in the CREATE registry reported that patients in the lower socio-economic strata (SES) were less likely to undergo coronary angiography, percutaneous coronary intervention, and coronary artery bypass graft surgery and were less likely to receive medications for secondary prevention following an episode of ACS. These disparities contributed significantly to the 2.7% absolute increase in 30-day mortality seen in the lowest SES versus the highest SES [22].

## PHARMACOLOGICAL MANAGEMENT OF HF

The drugs most commonly used for the management of HF are BB, ACEI (Angiotensin-converting enzyme inhibitors)/ARB (angiotensin receptor blockers), Diuretics and Aldosterone antagonists. Even in the developed world, medications are under-used [56]. The EuroHeart survey demonstrates that only 17.2% of HF patients were under the combination of a diuretic, ACEI and beta-adrenoceptor blocker, indicating the underuse of a life prolonging and a potentially cost-effective therapy [57]**.**

Affordability of medications is another major issue. In Argentina, China, India and Tanzania, Huffman and colleagues found that 10-12% of the patients do not take any medications following an episode of ACS due to inability to afford medications [54]. HF usually requires lifelong medications. Many of these patients are from low SES, for whom, affordability and compliance is a major hurdle.

As Bundkirchen and Schwinger [51] have noted, repeated hospitalizations are not only a powerful marker of poor prognosis and of poor life quality but they also lead to increased costs to the health care system. They reports that 75% of the direct costs are mainly attributed to hospitalizations. In the EuroHeart Failure survey programme, 24% of patients had been readmitted to hospital within 12 weeks of discharge [58].

The three main causes of hospitalizations due to chronic HF are sodium retention (55% of cases), ischaemic episodes or myocardial infarction (25%) and arrhythmia (15%) [59]. In view of this fact, outpatient management programs monitoring weight of the patient and adjusting the fluid intake may be a suitable strategy for managing the burden of HF. This can be done by trained nurses over telephone, called structured telephonic support [60].

## NON-PHARMACOLOGICAL MANAGEMENT OF HF

HF patients are at risk of sudden cardiac death (SCD) due to ventricular arrhythmias. Implantable Cardioverter Defibrillators (ICD) is indicated in patients with chronic HF at risk for SCD due to ventricular tachycardia or ventricular fibrillation. Similarly Cardiac Resynchronisation therapy (CRT) is now established in the treatment of patients with HF and evidence of intraventricular conduction delay in the ECG. In SA, a few centers implant these devices, but these facilities are concentrated in the major cities. The costs of these devices and their subsequent replacements are almost non-affordable to the vast majority of patients with HF in the region.

Management of end-stage HF with ventricular assist devices (VAD) and cardiac transplantation are undertaken in very few centers in the region. For example, the number of cardiac transplants performed so far in India is less than 100 and implant of VADs is limited to a handful of centres. Use of such devices is unlikely to be cost-effective when we are addressing a much bigger target population who need to be managed with lesser resources.

## PREVENTION OF HF IN SOUTH ASIA

HF is becoming a major burden to the health system in SA and it is likely to increase in the coming decades. Managing these patients will be an enormous task. So the better option will be to prevent the occurrence of HF. To reduce the burden of HF we have to reduce the prevalence of risk factors for HF, namely, Hypertension, diabetes, smoking and obesity.

Most important of the factors is hypertension. Landmark hypertension trials such as Swedish Trial in Old Patients with Hypertension (STOP) [61], Systolic Hypertension in the Elderly Program (SHEP) [62], and Systolic Hypertension in Europe (Syst-Eur) [63] demonstrated a 1.5%-2.5% absolute risk reduction in the incidence of HF over the 2-4 year follow up period with antihypertensive therapy [3].

Public awareness about hypertension needs to increase. Periodic health check-ups by grass-roots level health workers who can detect patients with hypertension and refer them for treatment as well as provide advice regarding salt reduction, regular physical activity and healthy diet patterns will significantly help in this regard. Legislation to limit the salt content of foods has great potential to reduce the burden of hypertension, CHD and subsequent incidence of HF across a wide spectrum of the population [3]. A 2010 study modeling a 3g reduction in salt intake across the population of USA estimated an annual reduction in myocardial infarction by 54 000-99 000, stroke by 32 000-66 000, and overall mortality by 44 000-92 000 [64]. Once hypertension is detected, optimal control of the blood pressure to target levels is very important to prevent the development of HF. Here we can use the services of primary health care workers to periodically check blood pressure to make sure that it is under control.

Tobacco control is the other most important strategy to reduce the burden of HF. Increasing the taxation of tobacco products and smokeless tobacco products will reduce the burden of CVD and thus HF. Banning smoking in public places have already shown the benefit of reduction in CVD [65].

South Asia already has one of the highest prevalence levels of diabetes mellitus (DM) in the whole world [66]. Analysis of the Framingham cohort revealed a 2.4 fold increase in the risk of symptomatic HF for diabetic men and five-fold risk of the same in diabetic women [67]. So this region is going to have increasing burden of HF due to DM.

Detection of DM in the community and controlling the blood sugar levels in the patients will reduce the burden of HF in the region. It is shown that strict control of all known risk factors for CVD and micro vascular complications by aggressive management of hypertension, dyslipidemia, and glycemia, use of aspirin, and cessation of smoking in patients with type 2 diabetes has proved to be highly beneficial in diabetic patients [68]. ACEIs, e.g. ramipril, are shown to reduce the incidence of HF in patients with diabetes as demonstrated in the Heart Outcomes Prevention Evaluation sub-study (MICRO-HOPE) [69]. Ramipril was found to reduce the incidence of HF by 2.3% over 5 years. So prescribing ACEI to diabetics will be beneficial in reducing the burden of HF. In addition, strategies aimed at preventing or delaying the onset of type 2 DM, e.g., intense lifestyle modifications or use of metformin could effectively reduce the burden of HF [6].

The prevalence of overweight and obesity are also increasing in the region [70]. Spreading the message of healthy diet and encouraging regular physical activity will help to reduce the burden of obesity.

Secondary prevention strategies in patients with established vascular disease with emphasis on regular follow-up, strict control of risk factors, life style modification and regular medications will reduce the incidence of HF. Patients with established atherosclerotic vascular disease need to be treated with statins. Statins have demonstrated reduction in the incidence of HF in patients with established atherosclerotic disease [71-73]. Statins also prevent the recurrence of events in these patients which itself will reduce the incidence of HF [73].

Congenital heart disease is another major contributor to the burden of HF in the region. Timely detection and referral for corrective procedures is essential to lessen the burden of HF in childhood and later in adult life. Many of the children in whom certain such defects are not timely attended to, go on to develop pulmonary vascular disease and HF in later age. Availability of facilities for the diagnosis and treatment of congenital heart diseases are limited to a few cities in the region and the numbers of trained pediatric cardiologists and pediatric cardiac surgeons are very few. Affordability of the treatments to correct these defects - either surgically or by percutaneous techniques to those poor people is also very limited. Establishing more centres, particularly in the public sector, and training more physicians to tackle these problems will reduce the burden. 

The rates of institutional delivery are improving in India (even if at a very slow pace), this gives a chance to detect heart disease in infancy itself which may reduce the burden of HF due to congenital heart disease. Pulse oximetry is an efficient way of screening infants and is endorsed recently by the American Academy of Pediatrics [74]. Support schemes where the surgeries of congenital heart diseases are done free-of-cost have been launched in many states in India (e.g.,“Thalolam” scheme in Kerala). Similar programs should be spread all over India and to the region.

Controlling RHD in the Indian subcontinent will require immense effort from various quarters. Improving the living standards by better housing and reducing over-crowding will reduce the incidence of rheumatic fever. Development of streptococcal vaccine has not reached any meaningful level and it may not be practical to be implemented worldwide due to variability in streptococcal strains [75]. Major reductions in RHD in Cuba [76] and Costa Rica [77] have been demonstrated through comprehensive programmes that increase community awareness of group A streptococcal infections and integrate clinical diagnostics and single dose benzathine penicillin treatment in primary care settings. While this strategy may not be easy to adopt throughout the region, it may be more cost-effective than secondary prevention alone [78].

Pulmonary hypertension is another major contributor to HF as we have seen. With the availability of vasodilator drugs like PDE V inhibitors and endothelin receptor antagonists there is some hope for these patients. Here too, timely detection and early initiation of treatment are important.

Affordability of medications is a problem in the treatment of HF and in the control of risk factors. Generic drugs can provide low-cost treatment to the majority of these patients who are in the low SES. Governments should encourage usage of generic drugs by physicians and also encourage distribution of these drugs through pharmacies.

To simplify the management of individuals who require medication for CVD, Wald and Law proposed a combination pill or polypill [79, 80]. Several versions of the polypill for treating cardiovascular disease have been successfully developed by pharmaceutical companies mainly from India. The combination of drugs is reported to be free of pharmacokinetic interactions between the ingredients, with preserved bioavailability [81]. The advantage of the polypills developed by these Indian companies are that their ingredients are generic drugs and they are expected to be affordable. A study which was done under the auspices of WHO (World Health Organization) is reported from Sri Lanka, where both the patients and treating doctors found the polypill to be safe and effective [82]. Polypills can be developed for patients with chronic HF along similar principles, which may be an option for this region.

## ORGANIZED HF PROGRAMS

As we have discussed earlier, a number of studies have documented substantial underuse of evidence-based guideline-recommended HF therapies and marked variation in the quality of care judged by specific performance measures and in patients receiving conventional care.

The traditional model of care delivery is thought to contribute to frequent hospitalizations in HF patients because in these brief, episodic encounters, little attention may be paid to the common, modifiable factors that precipitate many hospitalizations. Moreover, patient behavioural factors, such as non-adherence to diet and medications, and economic and social factors frequently contribute to disease progression, frequent hospitalizations, and diminished quality of life [83-85].

A number of HF disease management programs centred on the HF clinic model of care have been developed and have provided assessments of their impact on quality of care and clinical outcomes. HF patients who were cared for in these programs were shown to have improved utilization of evidence-based therapies, significantly fewer re-hospitalizations, lower health-care costs, improved functional and symptom status, and better quality of life compared with HF patients treated with conventional care [83-85]. A meta-analysis of 30 trials confirms that, multidisciplinary interventions for heart failure reduce both hospital admission and all-cause mortality rates [86]. HF clinic and non-clinic disease management interventions directed at recently hospitalized patients with HF significantly reduce re-hospitalizations and health-care costs with a trend toward lower all-cause mortality rates [86-87].

As yet, HF has not gained acceptance in the region as a subspecialty of cardiology. There are no specialised HF clinics even in major teaching institutions. Establishing dedicated HF clinics will be one way to tackle this problem. Dedicated HF rehabilitation programmes have been found successful in Indian settings also even though in small number of patients [88]. Imparting training and providing continued medical education to physicians will improve the management of HF patients. Participation in practice improvement programmes has been shown to increase use of evidence-based care, adherence to performance measures, and decreased length of stay (for hospitalized HF patients) and may improve clinical outcomes [89, 90].

Non-physician based follow-up programs have been found successful in follow-up of patients with HF. In SA region where the numbers of physicians are limited, such programs may benefit patients with HF [91].

## RESEARCH

There are no published data on any systematic study of HF from this region. There is a tremendous need for large multi-centre representative observational studies to quantify the disease burden, and to identify the aetiology of HF in the region. Hospital based registries can provide data about natural history and practice patterns in HF in the region. We require community-based studies to obtain the prevalence and incidence of HF in the region, and professional associations and medical universities can take the lead in these endeavours.

There is also lack of cardiovascular clinical practice guidelines, as well as nationally representative quality improvement initiatives to improve care for HF. Development of guidelines and quality improvement programmes through professional societies offers a potential avenue for clinicians and researchers to improve prevention of HF through the establishment and implementation of region/country-specific practice standards [3].

## CONCLUSION

Heart failure most likely contributes to significant disease burden in South Asia. With the increasing prevalence of risk factors in the region the burden of HF is likely to rise in the coming years. The aetiology of HF in the region is different from the west with RHD and CHD contributing significantly to the burden. The rising burden of HF is likely to impact the economy of the region in a big way. Accessibility and affordability to various treatment modalities is a major issue in this region. Prevention of HF assumes prime importance in this scenario. Controlling the risk factors - mainly hypertension, smoking and diabetes mellitus and treating heart diseases optimally will prevent the burden of HF significantly. Establishing HF clinics and initialising nurse and primary health care worker based management programs will help in reducing the disease burden. Research programs to generate data from the region and physician training programs will help in improving the management of HF in the region.

## Figures and Tables

**Table 1.** Shows the Aging of the Population in South Asia in Thousands. (Ref. http://esa.un.org/wpp/Excel-Data/population.htm, accessed July 18, 2012)

	Total Population	>60 Population	Total Population	>60 Population
	2011	2011	2051	2051
**Bangladesh**	151574	11160	200450	50724
**Bhutan**	744	58	992	268
**India**	1250232	104984	1742630	376722
**Maldives**	322	24	427	152
**Nepal**	30637	2089	47665	9085
**Pakistan**	177836	12486	281020	49629
**Sri Lanka**	21367	2974	24583	7836
**SOUTH ASIA**	1632713	133775	297767	494417
**% of Total Population**		8.2%		21.50%

**Table 2.** Prevalence Estimates of HF in India. (Huffman and Prabhakar) [3]

Condition (a)	Prevalence as in 2000	Incidence of HF Per Year	Prevalence of HF Attributed to Condition (a) After 5 Years
CHD	3% [3, 9]	0.4-2.3% [10, 11]	0.3-1.75 million
Hypertension	118 million	0.1-0.6%*	0.3-1.8 million
Diabetes	32 million [12]	2.3/1000 person years	0.18 million
Obesity (>30 Kg/m2)	5% [13]	0.3-0.5[14]	0.45-0.75 million

CHD- coronary heart disease, HF-heart failure.

* with a systolic blood pressure (SBP) of 144-154 mmHg [HOT[15] and UKPDS[16] trials]

**Table 3.** Frequency of Various Etiologies in Patients who were Admitted with HF in 2011 in SCTIMST, a Tertiary Cardiology Care Centre in India

Etiology	Frequency
Ischaemic Left ventricular dysfunction (with non-viable myocardium)	22
Dilated cardiomyopathy	20
Rheumatic heart disease	13
Infective endocarditis	6
Post valve surgery	8
Mitral valve prolapse	3
Pulmonary vascular disease	5
Aortic valve disease	5
HF with preserved ejection fraction	5
Peripartum Cardiomyopathy	2
Endomyocardial fibrosis	6
Aortoarteritis	2
Hypertrophic cardiomyopathy / restrictive cardiomyopathies	6
Constrictive pericarditis	2
Total	105

**Table 4.** Features of HF in South Asians versus Whites [30]

	South Asians versus Whites	Study
**Prevalence**	Similar	Galasko*, et al.* [31]
**Age of presentation **	Lower	Blackledge*, et al.* [32], Singh and Gupta [33]
**Ischaemic etiology of HF**	More common	Galasko*, et al.* [31]
**History of MI prior to the first HF admission**	Higher	Blackledge*, et al.* [32]
**Diabetes in HF patients**	More common	Blackledge*, et al.* [32]
**Atrial fibrillation in HF patients**	Less common	Newton*, et al.* [34]
**Hospital readmissions**	More common	Blackledge*, et al.* [32], Newton*, et al.* [34] Sosin*, et al.* [35]
**Age adjusted mortality**	Lower	Blackledge*, et al.* [32]
HF - Heart failure, MI - Myocardial infarction		

Published with permission from John Wiley and Sons.
